# The Influence of Culture Capital, Social Security, and Living Conditions on Children’s Cognitive Ability: Evidence from 2018 China Family Panel Studies

**DOI:** 10.3390/jintelligence10020019

**Published:** 2022-03-25

**Authors:** Xianhua Dai, Wenchao Li

**Affiliations:** 1 School of Public Administration, Central China Normal University, Wuhan 430079, China; liwenchao@mails.ccnu.edu.cn; 2 Center for Labor and Social Security Research, Central China Normal University, Wuhan 430079, China

**Keywords:** culture capital, economic capital, social capital, social security, living conditions, cognitive ability, heterogeneity

## Abstract

The aim of this study was to analyze the influence of economic capital, culture capital, social capital, social security, and living conditions on children’s cognitive ability. However, most studies only focus on the impact of family socio-economic status/culture capital on children’s cognitive ability by ordinary least squares regression analysis. To this end, we used the data from the China Family Panel Studies in 2018 and applied proxy variable, instrumental variables, and two-stage least squares regression analysis with a total of 2647 samples with ages from 6 to 16. The results showed that family education, education expectation, books, education participation, social communication, and tap water had a positive impact on both the Chinese and math cognitive ability of children, while children’s age, gender, and family size had a negative impact on cognitive ability, and the impact of genes was attenuated by family capital. In addition, these results are robust, and the heterogeneity was found for gender and urban location. Specifically, in terms of gender, the culture, social capital, and social security are more sensitive to the cognitive ability of girls, while living conditions are more sensitive to the cognitive ability of boys. In urban locations, the culture and social capital are more sensitive to rural children’s cognitive ability, while the social security and living conditions are more sensitive to urban children’s cognitive ability. These findings provide theoretical support to further narrow the cognitive differences between children from many aspects, which allows social security and living conditions to be valued.

## 1. Introduction

This study analyzed the influence of economic capital, culture capital, social capital, social security, and living conditions on children’s cognitive ability.

With deepening development and reformation of education, the human capital cultivation of children is becoming a key step for many families. A fundamental aspect of the cultivation is children’s cognitive ability, which is the ability of human beings to extract, store, and use information from the objective world. It mainly involves human abstract thinking, logical deduction, and memory (Autor 2014). As documented, there is a significant correlation between family factors and children’s cognitive ability (Zimmer et al. 2007; Kleinjans 2010; Li 2012, 2017; Saasa 2018; Fan et al. 2019; Wang and Lin 2021). Specifically, there are three different capital theories that focus on the impact of family on children’s cognitive ability, namely economic capital, cultural capital, and social capital (Bourdieu and Wacquant 1992; Farkas 2003). In particular, the impact of cultural capital is particularly important (Li and Zhao 2017; Yao and Ye 2018; Zhang and Su 2018; Hong and Zhang 2021) since economic capital reflects its value only by cultural capital (Hong and Zhao 2014). In addition, there are great differences in economic capital, social capital, and cultural capital between urban and rural families, which leads to the urban–rural education gap (Jin 2019). These findings concentrate on the influence of family capital on children’s cognitive ability, but the social security and living conditions are not touched upon. In contrast, this study investigated the influence of all those factors on children’s cognitive ability, in particular, the social security and living conditions.

Different family capital has corresponding measurement indicators. In particular, economic capital includes family income (Yang and Wan 2015; Fang and Hou 2019; Hou et al. 2020), health investment (Shen 2019; Wu et al. 2021), and education expenditure (Lin et al. 2021; Fang and Huang 2020), which refers to the sum of economic related resources owned by a family (Xue and Cao 2004). Culture capital is not only reflected in the diplomas obtained by family members, but also in the educational concept, attitude, and expectation of parents for their children (Guo and Min 2006), which includes three forms: concrete culture capital, such as family parenting (Zhang et al. 2017; Huang 2018), lifestyle (Wu et al. 2020), education expectation (Gu and Yang 2013; Wang and Shi 2014; Xue 2018; Zhou et al. 2019), participation (Wei et al. 2015; Liu et al. 2015; Liang et al. 2018); objectified culture capital, including books (Hong and Zhao 2014; Yan 2017); and institutionalized culture capital, referring to the educational diploma obtained (Xie and Xie 2019; Zhu et al. 2018). From the perspective of micro social network, social capital referred to in this paper is defined as a kind of resource embedded in the network (Granovetter 1973), which takes social capital as a new form of capital, so that actors can obtain a better professional position or business opportunities, so as to affect the income return (Lin 2005). In specific, social capital includes occupation (James 2000; Teacherman 2000; Fang and Feng 2005; Zhou and Zou 2016; Zhu and Zhang 2020), social communication (Putnam 2000; Liang 2020; Yang and Zhang 2020), information utilization (Cao et al. 2018; Zheng et al. 2021), and human expenditure (Wang and Gong 2020).

Social security can improve residents’ household consumption (Fang and Zhang 2013; Yang and Yuan 2019) and alleviate economic poverty (Guo and Sun 2019) through income redistribution, which can increase the economic capital of families and affect investment in children. Thus, the social security affects children’s cognitive ability, including medical insurance (Chen et al. 2020), endowment insurance (Xue et al. 2021), and government support (Liu and Xue 2021; Yin and Fan 2021). Living conditions refer to the family infrastructure and facilities that affect children’s lives, including safe drinking water, sanitary toilets, clean energy, waste treatment, and sewage treatment (Zhao et al. 2018). In particular, exposure to air pollution (Chen et al. 2017a; Schikowski and Altug 2020; Nauze and Severnini 2021), water (Chen et al. 2017b; Gao et al. 2021), and fuel (Cong et al. 2021; Chen et al. 2021) also affects cognitive ability. Other factors include family structure (Zhang 2020; Jiang and Zhang 2020), family size (Liu and Jin 2020; Fang et al. 2020), and family health (Li and Fang 2019). Unlike previous work, this study applied instrumental variables and two-stage least squares regression analysis to solve the endogenous problem, assessing the influence of numerous factors on children’s cognitive ability. The robustness of this study’s results was assessed by controlling sample size and increasing variables.

In addition, children’s individual and social characteristics affect cognitive ability. For example, the performance of girls is better than that of boys, although the gender difference is decreasing (Hao 2018). The older the migrant child, the worse the academic performance (Wang and Chu 2019). Number of siblings has a significant impact on youth’s cognitive ability (Tao 2019). In contrast, this study investigated heterogeneity in gender and urban location for those influences.

This study examined the impact of numerous factors, including social security and living conditions, on children’s cognitive ability, using data from the China Family Panel Studies in 2018. Rather than the ordinary least squares method, the study used two-stage least squares regression to solve endogeneity. In addition, we explored heterogeneity in gender and urban location and the impact of those factors on children’s cognitive ability. These results obtained may provide guidance for the government, society, and families to improve children’s cognitive ability.

The remainder of this paper is organized as follows. Section 2 describes data, variables, and summary statistics. Section 3 outlines the basic model for the influence of those factors on children’s cognitive ability. Section 4 describes the instrumental variable test, endogeneity test, empirical results, and robustness test. Section 5 outlines the heterogeneity analysis of gender and urban location. Section 6 concludes.

## 2. Data, Variable, and Summary Statistics

### 2.1. Data

This study used the data from the China Family Panel Studies (CFPS), a tracking survey of individuals, families, and communities implemented by China Social Science Investigation Center of Peking University, which aims to reflect the changes of China’s society, economy, education, and health. The data sample covers 25 provinces/cities/autonomous regions, and the respondents include all family members. In the implementation of the survey, the multi-stage, implicit stratified, and population scale proportional sampling method was used. The main research object of this study was children aged 6–16. Since the respondents of the CFPS personal self-administered questionnaire are children over nine years old, and children’s cognition of their own situation is not necessarily accurate, this study mainly used the children’s proxy questionnaire and combined the relevant variables such as parents’ situation in the personal self-administered questionnaire and family basic information in the family questionnaire. The data supported this work. The basic information related to families, parents, and their children in 2018 was extracted and matched with the data.

### 2.2. Explained Variables

Following Li and Shen (2021), Wu et al. (2020), and Dong and Zhou (2019), children’s Chinese and math scores were used in this study to measure Chinese cognitive understanding ability and math reasoning cognitive ability, respectively, using the “How about Chinese score” and “How about math score” tests in the CFPS questionnaire, both of which use ordinal categorical variables (1 for “fail”, 2 for “intermediate”, 3 for “good”, and 4 for “distinction”).

### 2.3. Explanatory Variables

In this study, the main explanatory variables were divided into five parts. They are economic capital, culture capital, social capital, social security, and living conditions.

Economic capital was measured by the family income, children’s health investment, and education investment. They are all continuous variables and were added 1 before taking the natural logarithm.

Culture capital was measured by the questions of “How many books do you have in your family?”, “What is the highest degree you have completed?”, “What level of education do you want your child to attain?”, “How often do you discuss what’s happening at school with your child?”, and “When your children’s grades are not satisfactory, which way do you usually deal with them?”. They represent the family books, education, educational expectation, educational participation, and parenting style, respectively. There are three aspects of culture capital, namely the objective, institutional, and concrete culture capital (Bourdieu and Passeron 1977). For family education and education expectation, 0 is for illiterate/semi-illiterate, 1 for nursery, 2 for kindergarten, 3 for primary school, 4 for junior middle school, 5 for senior middle school, 6 for junior college, 7 for undergraduate, 8 for master, and 9 for doctor. For parenting style, we redefined scolding the child, spanking the child, and restricting the child’s activities as 0, and contacting the teacher, telling the child to study harder, helping the child more, and doing nothing as 1. Among them, 0 is for stern parenting, and 1 is for gentle parenting. Family books and children’s education participation are continuous variables, and the number of books was added 1 before taking the natural logarithm. In addition, family lifestyle consists of smoking, drinking, exercise, and lunch break, which is an ordered variable.

Social capital was measured by “nature of work”, “information utilization”, “social communication”, and “human expenditure”. For job, 1 is unemployed, 2 is agricultural work, and 3 is non-agricultural work. We used the questions of “Do you use a mobile phone?”, “Do you use mobile devices?”, and “Do you use a computer to surf the Internet?” to measure the information utilization. We defined information utilization as follows: 0 means that none is used, 1 means that at least one is used, 2 means that at least two are used, and 3 means that at least three are used. The questions of “How good do you think your relationship is?” and “How do you rate your trust in your neighbors?” were used to measure the social communication. We summed and then averaged the answers to these two questions and obtained a continuous variable. Human expenditure is a continuous variable and was added 1 before taking the natural logarithm.

Social security was measured by the participation of medical and endowment insurance and government support. Among them, medical and endowment insurance are continuous variables. For government support, 0 is for not accepting subsidies, and 1 is for accepting the subsidies.

Living conditions were measured by the questions about “water for cooking”, “cooking fuel”, and “indoor air purification”, and the answer 0 is for no and 1 is for yes. Specifically, for tap water, 0 represented no tap water use, and 1 is for tap water use. For cooking fuel, 0 is for no use of clean fuel, and 1 is for clean fuel use. For air purification, 0 is for no air purification, and 1 is for use of air purification. In addition, for gender, 0 is for women and 1 is for men. The registered residence was redefined: 0 is for rural, and 1 is for urban. The registered marital status was redefined: 0 is for unmarried, and 1 is for married. For nationality, 0 is for others, and 1 is for Han nationality. Family age, the child’s age, and family size are the continuous variables. For family health, 1 denotes unhealthy, 2 relatively unhealthy, 3 average, 4 relatively healthy, and 5 very healthy. We used the question “How many times a week do you eat with your family?” to measure parenthood, which is a continuous variable.

In addition, we consider parents’ cognitive ability as proxy variable of genes. According to the CFPS in 2018 for the children’s questionnaire, the respondents may be father or mother. Following Li and Zhang (2018), we select two dimensions of father’s or mother’s word ability and mathematical ability to construct parents’ cognitive ability indicators. To compare, we standardized the scores of word ability and mathematical ability, and added up to obtain a comprehensive cognitive ability, which is recorded as family cognitive ability.

Table 1 shows the summary statistics of variables.

By deleting invalid values, 2647 final valid samples were included. As shown in Table 1, for children’s characteristics, approximately 54% of children were boys, 46% were girls, 43% lived in urban areas, 57% lived rurally, and the children’s age ranged from 6 to 16. For family characteristics, approximately 35% were male, 65 were female, 96% had a spouse, the family age ranged from 18 to 78, and the average family size was 5.

For family economic capital, the mean values of family income, children’s health investment, and education investment are 10.74, 4.30, and 7.27, respectively. Education investment is significantly greater than health investment. For family culture capital, approximately 89% of families adopted a mild parenting approach, the frequency of families talking with their children is 3.26, the average educational level of the family is primary school, and the family education expectation is undergraduate. The average value of family lifestyle is 1.87, indicating that families account for at least two of smoking, drinking, exercise, and lunch break. The average number collected books in the family is 2.51. Institutionalized and materialized cultural capital are not high, but the level of morphological cultural capital is relatively high, indicating that families pay more attention to education.

For family social capital, family non-agricultural employment is significantly greater than agricultural employment or unemployment; the average family information and human expenditure are 1.79 and 7.372, respectively; the popularity of social communication is 6.83; and the family social capital is moderate to good. For family social security, every family has at least one kind of medical insurance and endowment insurance, and at least half of the people have received government subsidies. For living conditions, the values for utilities of tap water, fuel, and air purification are 73%, 70%, and 3%, respectively; the popularity of tap water and clean fuel is high, while the popularity of air purifiers is low. In addition, children’s Chinese and math cognitive ability were both moderate; the average cognitive ability of math is higher than that of Chinese.

For family cognitive ability, the average of Chinese and math cognitive ability is 18.33 and 8.74, respectively, and the overall level of family cognitive ability is not high. We included the standardized and aggregated comprehensive family cognitive ability in Table 1, with a maximum of 4.70 and a minimum of −3.53.

## 3. Basic Model

This study included 29 characteristics as covariates. To investigate effect of those factors on children’s Chinese cognitive ability and math cognitive ability, respectively, we established the following model.

(1) $$E_{ni}=\beta_{0}+\sum_{k=1}^{3}\beta_{k1}C_{ki}+\sum_{j=1}^{6}\beta_{j2}F_{ji}+\sum_{l=1}^{20}\beta_{l3}S_{li}+\varepsilon_{i}$$

where $E_{ni}$ is the *n*-th cognitive ability for the child *i* (*n* = 1, 2, where 1 is for Chinese and 2 for math); $C_{ki}$ is the *k*-th children’s characteristics for the child *i* (*k* = 1, 2,…3); $F_{ji}$ is the *j*-th family information for the child *i* (j = 1, 2,…, 6); $S_{li}$ is the *l*-th family capital and family cognitive ability for the child *i* (*l* = 1, 2,…, 20); $\beta_{k1}$, $\beta_{j2}$, and $\beta_{l3}$ are the corresponding parameters to those variables, and $\varepsilon_{i}$ is the regression error term.

Through the above model, we used ordinary least squares (OLS) regression to obtain results. However, due to the reverse causal relationship and confounding factor, we had to find proxy variable to genetic, instrumental variables to solve endogeneity, and verify them according to the assumptions. Thus, we used two-stage least squares (2SLS) as the main empirical approach and compared with ordinary least squares (OLS). As a robustness check, we conducted analysis by adding variables and controlling sample size. In addition, the heterogeneity in gender and urban location was checked based on two-stage least squares (2SLS).

As for the sharing genes and environment between parents and children being concerned, we make the following discussion. On the one hand, the social environment experienced by children and their parents is different. In specific, the children studied in this paper were born in the 21st century, so they did not experience major social changes and disasters. However, their parents have experienced great social changes, for example, cultural revolution, educational reform, and natural disasters. On the other hand, the inequality of family resources will lead to the inequality of children’s cognitive ability and early skills dependent partly on genetics (Plomin and von Stumm 2018; Silventoinen et al. 2020). Thus, these two factors usually produced an interesting phenomenon, that is, the higher the importance of one, the smaller the other. However, as resulted by Houmark et al. (2020), the relative importance of genes depends on how parents’ investment is distributed among their children, whether parents or society are. As also resulted by Victor Ronda et al. (2020), the worse the childhood environment, including family resources, the weaker the role of their genes. In addition, as proved, cognitive ability can be developed through acquired cultivation (Hu and Xie 2011; Kuang et al. 2019; Zhou et al. 2021), but the cognitive ability, in this paper, refers to children’s word understanding ability and mathematical reasoning ability, which are measured by the scores of Chinese and math tests, respectively, and not measured by IQ test scores, though IQ test scores largely depend on genes. Furthermore, as observed from the samples in CFPS data, Chinese and math cognitive abilities of children with the same family ID were inconsistent. In particular, since the data of the 2018 China Family Panel Studies that we applied in this work do not provide genetic information, we take parents’ cognitive ability as the proxy variable of genes in regression analysis.

In this study, proxy variables meet the following two conditions: (1) After introducing proxy variables (parental cognitive ability), there is no correlation between family capital and genes. Indeed, following Zheng et al. (2018), family capital is an acquired environmental factor. (2) Once the genes are observed, parents’ cognitive ability will no longer mainly explain children’s cognitive ability. Specifically, parental cognitive ability is highly correlated with their genes, and parental cognitive ability is not collinear with other explanatory variables. As checked, parental cognitive ability is not related to random error, and family cognitive ability can be used as a proxy variable to reflect the genetic difference.

Following Cui and Susan (2022), instrumental variables and two stage least squares regression are applied. In particular, when the exposed group and the non-exposed group are not comparable, some background variables need be used to stratify the total group so that the exposed sub-group and the non-exposed sub-group are comparable. Instrumental variable analysis can control those bias in observational studies (Geng 2004; Brookhart et al. 2006). The instrumental variables and two stage least squares analysis in this paper will be shown in Section 4.2.

## 4. Results

### 4.1. Results from OLS

Using the survey data of CFPS in 2018, we successively incorporated family cognitive ability and family capital into the regression and applied the ordinary least squares (OLS) method to investigate the influence of family economic capital, culture capital, social capital, social security, living conditions, and family cognitive ability on children’s Chinese and math cognitive ability. After excluding the influence of collinearity, the results are shown in the second to fifth column of Table 2.

As shown in second and third columns of Table 2, the effect of family cognitive ability on children’s cognitive ability was significant (0.054, *p* < 0.01), i.e., the shared genes partly determine children’s cognitive ability. As shown in the fourth and fifth columns of Table 2, the effect of family cognitive ability is no longer significant, i.e., the role of genes will be weakened by family capital. This has also been confirmed in Victor Ronda et al. (2020). Besides, children’s age (−0.053, *p* < 0.01) and gender (−0.287, *p* < 0.01) have significant influence on Chinese cognitive ability, while only children’s age (−0.096, *p* < 0.01) has significant influence on math cognitive ability. The influence of children’s age and gender on the two cognitive abilities are both negative, while family age (0.005, *p* < 0.05; 0.007, *p* < 0.01) has a positive effect on their children’s cognitive ability for Chinese and math.

For family culture capital, family education (0.081, *p* < 0.01; 0.085, *p* < 0.01), education expectation (0.122, *p* < 0.01; 0.163; *p* < 0.01), and family books (0.020, *p* < 0.1; 0.019, *p* < 0.1) have a positive impact on the two cognitive abilities. Among them, education expectation has the greatest impact, followed by family education and family books, and the influence of education expectation and family education on math cognitive ability is greater than that of Chinese, while the influence of family books is opposite. The more frequently families participate in education (0.082, *p* < 0.01; 0.058, *p* < 0.01), the better their children’s cognitive abilities, and the impact on Chinese cognitive ability is greater than the impact on math. For family social capital, the impact of social communication on both children’s Chinese (0.045, *p* < 0.01) and math (0.038, *p* < 0.01) cognitive abilities is positive. For living conditions, only tap water (0.089, *p* < 0.05) exhibited a positive impact on children’s Chinese cognitive ability. In general, cultural capital has the greatest impact, followed by living conditions and social capital. However, the influence of family economic capital is not significant. The above results are based on ordinary least squares (OLS).

### 4.2. Endogeneity Test

In Equation (1), to avoid the endogenous problems caused by omitted variables, we consider the children’s characteristics and family information, including age, gender, nationality, residence, marriage, and family size. These variables have been proved to have an impact on children’s cognitive ability in previous studies. In this model, the main endogenous problems may be caused by the confounding factors and mutual causality. For example, children of high cognitive ability may have better genes than those of low cognitive ability. If children of high cognitive ability do not receive the acquired training, they are also more likely to obtain high cognitive ability, since their genes are excellent. However, as summarized by Miettinen and Cook (1981), confounding factors are independent risk factors; the distribution of confounding factors in exposed population and non-exposed population is different. So, we take family cognitive ability as poxy variable of genes.

Family books and family medical insurance passed the test of endogenous variables, while the family cognitive ability did not. Possible causes are confounding factors or mutual causality. For mutual causality, family books and family medical insurance may affect children’s cognitive ability. Conversely, children of higher cognitive ability may have more books bought for them by their parents to support and encourage them, and the medical insurance decision will also change (Zhang and Li 2021). Therefore, we solve these problems by selecting appropriate instrumental variables. Specifically, we adopted instrumental variables (IVs) and two-stage least squares (2SLS). We used the lag variable Bookiv as the instrumental variable of family books and the average participation rate of medical insurance (Mediv) in 28 provinces as the instrumental variable of medical insurance.

Our instrumental variables satisfy the assumptions of IVs (Angrist et al. 1996). Specifically, Bookiv is highly correlated with family books, and its impact on children’s cognitive ability is realized through family books, rather than directly affecting children’s cognitive ability. For Mediv, which is highly correlated with family medical insurance, the average participation rate does not have a direct impact on children’s cognitive ability. No other confounding factors exist between instrumental variables and children’s cognitive ability. In the previous literature, the factors that affect children’s cognitive ability were included in the regression to avoid the influence of confounding factors. To ensure that the IV estimation was reliable, we used the weak instrumental variable test, and as the result show, family books and medical insurance are endogenous variables. Furthermore, the Cragg–Donald–Wald F is 30.984, which is obviously greater than 10.

As shown in sixth and seventh columns of Table 2, children’s age (−0.055, *p* < 0.01) and gender (−0.284, *p* < 0.01) have significant influence on their Chinese cognitive ability. The influence of children’s age and gender on the two cognitive abilities is negative, while the influence of family age (0.006, *p* < 0.05; 0.008, *p* < 0.01) is positive. For family culture capital, family education (0.087, *p* < 0.01; 0.090, *p* < 0.01), education expectation (0.116, *p* < 0.01; 0.158; *p* < 0.01), and books (0.101, *p* < 0.05; 0.089, *p* < 0.1) have a positive impact on the two cognitive abilities. Similarly, education expectation has the greatest impact, followed by family education and books, and the influence of education expectation and family education on math cognitive ability is greater than that of Chinese, respectively, while the influence of family books is the opposite. The more frequently families participate in education (0.078, *p* < 0.01; 0.055, *p* < 0.01), the better their children’s cognitive abilities, and the impact on Chinese cognitive ability is greater than on math. For family social capital, the impact of social communication on both children’s Chinese (0.048, *p* < 0.01) and math (0.039, *p* < 0.01) cognitive abilities is positive. In addition, for family social security, medical insurance (−1.427, *p* < 0.01; −1.273, *p* < 0.01) has negative impact on both Chinese and math cognitive abilities, while endowment insurance (0.229, *p* < 0.01; 0.183, *p* < 0.05) has positive impact on both Chinese and math cognitive abilities. Tap water (0.091, *p* < 0.1) has a positive impact on children’s Chinese cognitive ability. After introducing instrumental variables, the impact of family books and medical insurance on children’s cognitive ability increased. The above results are based on the two-stage least squares (2SLS).

### 4.3. Robustness Checks

To verify the reliability of the estimated results, we carried out robustness checks using three methods. Specifically, we controlled the sample size and the number of explanatory variables and took the family health and family relationship into account. Family health refers to the self-evaluation of family health: 1 for unhealthy and 5 for healthy. Family relationship is a continuous variable measured by the number of meals with family members.

As shown in the second and third columns of Table A1 in Appendix A, children’s age (−0.055, *p* < 0.01; −0.098, *p* < 0.01), children’s gender (−0.284, *p* < 0.01, for Chinese), family age (0.007, *p* < 0.05; 0.009, *p* < 0.01), family education (0.086, *p* < 0.01; 0.089, *p* < 0.01), education expectation (0.115, *p* < 0.01; 0.157, *p* < 0.01), books (0.105, *p* < 0.05; 0.093, *p* < 0.05), education participation (0.076, *p* < 0.01; 0.054, *p* < 0.01), social communication (0.044, *p* < 0.01; 0.035, *p* < 0.01), medical insurance (−1.450, *p* < 0.01; −1.287, *p* < 0.01), endowment insurance (0.236, *p* < 0.01; 0.188, *p* < 0.05), and tap water (0.092, *p* < 0.05, for Chinese) still have significant influence on children’s cognitive ability. Family health (0.038, *p* < 0.05; 0.041, *p* < 0.05) has a positive impact on the two cognitive abilities. Similarly, as shown in the fourth, fifth, sixth, and seventh columns in Table A1 in Appendix A, the significance remains unchanged. Therefore, the results based on 2SLS are robust.

## 5. Heterogeneity Analysis

The heterogeneity was checked to determine the influence of family factors on children’s Chinese and math cognitive abilities.

### 5.1. Heterogeneity in Gender

As shown in Table A2 in Appendix A, for family culture capital, the influence of family education (0.100, *p* < 0.01; 0.102, *p* < 0.01, for girls) and education participation (0.133, *p* < 0.01; 0.104, *p* < 0.01, for girls) on girls’ cognitive ability is greater than that of boys. The influence of family education expectation on girls’ (0.157, *p* < 0.01) Chinese cognitive ability is greater than that of boys (0.093, *p* < 0.01), while the influence of family education expectation on boys’ (0.162, *p* < 0.01) math cognitive ability is greater than that of girls (0.157, *p* < 0.01). Family books (0.135, *p* < 0.1) only have a significant impact on girls’ Chinese cognitive ability. For family social capital, social communication has the greatest impact on girls’ cognitive ability (0.054, *p* < 0.01; 0.049, *p* < 0.05, for girls). For social security, medical insurance (−1.958, *p* < 0.05; −1.619, *p* < 0.05, for girls) and endowment insurance (0.298, *p* < 0.05; 0.271, *p* < 0.05, for girls) have the greatest impact on girls’ cognitive ability. For living conditions, only tap water has a positive impact on boys’ math cognitive ability (0.145, *p* < 0.05). In addition, the larger the family size, the greater the impairment of boys’ math cognitive ability. Therefore, the culture capital, social capital, and social security are more sensitive to girls’ cognitive ability, while living conditions are more sensitive to boys’ cognitive ability.

### 5.2. Heterogeneity in Urban Location

As shown in Table A3 in Appendix A, for family culture capital, the influence of family education on the cognitive ability of rural children (0.101, *p* < 0.01; 0.116, *p* < 0.01) is greater than that of urban children (0.065, *p* < 0.05; 0.069, *p* < 0.05). Family education expectation has the greatest impact on rural children’s math cognitive ability (0.191, *p* < 0.01) and urban children’s Chinese cognitive ability (0.123, *p* < 0.01). Family books only affects the math cognitive ability of urban children (0.108, *p* < 0.1). Family education participation has the greatest impact on rural children’s Chinese cognitive ability (0.092, *p* < 0.01) and the least impact on urban children’s Chinese cognitive ability (0.054, *p* < 0.1). For social communication, the impact on the cognitive ability of rural children (0.057, *p* < 0.01; 0.039, *p* < 0.05) is greater than that of urban (0.041, *p* < 0.05; 0.035, *p* < 0.1). Medical (−1.468, *p* < 0.01; −1.087, *p* < 0.05) and endowment insurance (0.243, *p* < 0.05; 0.193, *p* < 0.1) have a significant impact on the cognitive ability of urban children but not on rural children. For living conditions, only tap water (0.149, *p* < 0.1) was significant for urban children’s Chinese cognitive ability. Therefore, the culture capital and social capital are more sensitive to rural children’s cognitive ability, while the social security and living conditions are more sensitive to urban children’s cognitive ability.

## 6. Conclusions

This study used the data from the 2018 China Family Panel Studies to analyze the impact of numerous factors on children’s Chinese and math cognitive ability.

Firstly, children’s and family’s characteristics have significant impact on children’s Chinese and math cognitive ability. Among them, children’s age, gender, and family size are negative for children’s cognitive ability, while family age has a positive impact on children’s cognitive ability. Family culture capital, education, education expectation, books, and education participation have a positive impact on children’s cognitive ability. For family social capital, the more family social communication, the higher children’s cognitive ability. For family living conditions, family use of tap water is more conducive to the improvement of children’s cognitive ability. What is more, the influence of family cognitive ability on children’s cognitive ability is attenuated by the family capital, which means that the impact of genes are weakened. The above results are based on ordinary least squares (OLS). After introducing instrumental variables Bookiv and Mediv and solving endogeneity, some changes took place in the results. On the one hand, the influence of family books on children’s cognitive ability increased significantly. On the other hand, the impact of medical insurance and endowment insurance on children’s cognitive ability became significant. Medical insurance was negative, and endowment insurance was positive. In addition, according to the two-stage least squares (2SLS) method, the results are robust after controlling the sample size and increasing the variables.

Moreover, there is heterogeneity in gender and urban location for the influence of numerous factors on children’s Chinese and math cognitive ability. In regard to gender, the culture capital, social capital, and social security are more sensitive to girls’ cognitive ability, while living conditions are more sensitive to boys’ cognitive ability. Specifically, girls’ family education, education expectation, books, education participation, social communication, and medical and endowment insurance have a greater impact on cognitive abilities, and tap water is significant for the math cognitive ability of boys. In urban locations, the culture capital and social capital are more sensitive to rural children’s cognitive ability, while the social security and living conditions are more sensitive to urban children’s cognitive ability. Specifically, rural children’s family education, education expectation, education participation, and social communication have a greater impact on cognitive ability, while urban children’s family books, medical insurance, endowment insurance, and tap water are more significant for their cognitive ability.

There are some open problems following this research. Due to the imbalance of the initial sample proportion, the proportions of agricultural residence and non-agricultural residence samples were slightly unbalanced after data processing. The heterogeneity in urban location may lead to a slight bias in our full sample model. The error terms of the model may not be independently identically distributed. In addition, there may be further heterogeneity for the influence of numerous factors on children’s Chinese and math cognitive ability, and a full mediation analysis should be worthwhile in the future. In this study, we take family cognitive ability as proxy variable of genes, but the empirical results reported in this study are worth checking in full data directly including genetics and environment.

Those findings above provide theoretical support to further narrow the cognitive differences between children.

## Figures and Tables

**Table 1:** Summary statistics of variables.

	Number	Min (M)	Max (X)	Average (E)	Standard Error	Standard Deviation	Variance
Chinese (understanding)	2647	1	4	2.760	(0.019)	0.978	0.956
Math (reasoning)	2647	1	4	2.790	(0.020)	1.041	1.083
Child’s age	2647	6	16	10.90	(0.049)	2.538	6.442
Child’s gender	2647	0	1	0.540	(0.010)	0.499	0.249
Child’s nationality	2647	0	1	1.000	(0.001)	0.043	0.002
Residence	2647	0	1	0.180	(0.007)	0.381	0.145
Urban–rural	2647	0	1	0.430	(0.010)	0.495	0.245
Family age	2647	18	78	41.66	(0.178)	9.181	84.288
Family gender	2647	0	1	0.350	(0.009)	0.477	0.228
Family marriage	2647	0	1	0.960	(0.004)	0.201	0.041
Family size	2647	2	15	5.260	(0.038)	1.978	3.912
Family income	2647	0	13.82	10.744	(0.021)	1.071	1.148
Family health investment	2647	0	11.37	4.304	(0.055)	2.814	7.917
Family education investment	2647	0	11.69	7.265	(0.035)	1.776	3.153
Family education	2647	0	8	3.450	(0.036)	1.834	3.363
Family books	2647	0	9	2.510	(0.038)	1.931	3.727
Family education expectation	2647	3	9	6.800	(0.019)	1.002	1.005
Family parenting	2647	0	1	0.890	(0.006)	0.312	0.098
Family education participation	2647	1	5	3.260	(0.022)	1.135	1.287
Family lifestyle	2647	0	4	1.87	(0.016)	0.802	0.643
Family occupation	2647	1	3	2.400	(0.012)	0.624	0.389
Family information	2647	0	3	1.790	(0.015)	0.753	0.566
Family human expenditure	2647	0	11.00	7.372	(0.042)	2.178	4.743
Family social communication	2647	1	10	6.830	(0.031)	1.583	2.505
Family medical insurance	2647	0	3	0.950	(0.006)	0.292	0.086
Family endowment insurance	2647	0	4	0.720	(0.011)	0.565	0.319
Family government support	2647	0	1	0.500	(0.010)	0.500	0.250
Tap water	2647	0	1	0.73	(0.009)	0.445	0.198
Fuel	2647	0	1	0.70	(0.009)	0.458	0.210
Air purification	2647	0	1	0.03	(0.003)	0.178	0.032
Family heath	2647	1	5	3.04	(0.023)	1.187	1.408
Family relationship	2647	0	7	6.20	(0.036)	0.851	3.425
Family Chinese cognitive ability	2647	0	34	18.33	(0.216)	11.121	123.676
Family math cognitive ability	2647	0	24	8.74	(0.096)	4.637	21.504
Family cognitive ability	2647	−3.53	4.70	0.00	(0.034)	1.729	2.990

**Table 2:** Results for the influence of many factors on children’s cognitive ability.

	Chinese (OLS) *N* = 2647	Math (OLS) *N* = 2647	Chinese (OLS) *N* = 2647	Math (OLS) *N* = 2647	Chinese (2SLS) *N* = 2647	Math (2SLS) *N* = 2647
Intercept term	3.736 *** (0.458)	4.317 *** (0.483)	1.903 *** (0.511)	2.181 *** (0.538)	2.968 *** (0.641)	3.088 *** (0.655)
Child’s age	−0.058 *** (0.008)	−0.103 *** (0.008)	−0.053 *** (0.008)	−0.096 *** (0.008)	−0.055 *** (0.008)	−0.098 *** (0.009)
Child’s gender	−0.287 *** (0.037)	0.000 (0.039)	−0.287 *** (0.036)	0.001 (0.038)	−0.284 *** (0.040)	0.004 (0.041)
Child’s nationality	−0.327 (0.426)	−0.635 (0.449)	−0.400 (0.416)	−0.698 (0.438)	−0.438 (0.459)	−0.729 (0.469)
Family age	0.003 (0.002)	0.004* (0.002)	0.005 ** (0.002)	0.007 *** (0.003)	0.006 ** (0.003)	0.008 *** (0.003)
Family gender	−0.038 (0.039)	−0.046 (0.042)	−0.036 (0.041)	−0.067 (0.043)	−0.028 (0.045)	−0.060 (0.046)
Residence	0.158 *** (0.055)	0.167 *** (0.058)	−0.004 (0.058)	−0.011 (0.061)	−0.129 * (0.074)	−0.117 (0.075)
Urban–rural	0.031 (0.042)	0.091** (0.044)	−0.044 (0.044)	0.026 (0.046)	−0.060 (0.049)	0.012 (0.050)
Family marriage	0.129 (0.093)	0.133 (0.098)	0.066 (0.091)	0.059 (0.096)	0.044 (0.101)	0.040 (0.103)
Family size	−0.024 ** (0.010)	−0.022 ** (0.010)	−0.016 (0.010)	−0.018 * (0.010)	−0.008 (0.011)	−0.012 (0.011)
Family cognitive ability	0.054 *** (0.012)	0.054 *** (0.012)	−0.015 (0.014)	−0.011 (0.015)	−0.022 (0.016)	−0.017 (0.016)
Family income			−0.002 (0.019)	0.020 (0.020)	−0.002 (0.022)	0.023 (0.023)
Children’s health investment			−0.004 (0.007)	−0.003 (0.007)	−0.003 (0.007)	−0.003 (0.007)
Children’s education investment			0.014 (0.011)	−0.001 (0.012)	0.013 (0.012)	−0.002 (0.013)
Family education			0.081 *** (0.015)	0.085 *** (0.015)	0.087 *** (0.017)	0.090 *** (0.017)
Family education expectation			0.122 *** (0.018)	0.163 *** (0.019)	0.116 *** (0.021)	0.158 *** (0.022)
Family books/Bookiv			0.020 * (0.010)	0.019 * (0.011)	0.101 ** (0.046)	0.089 * (0.047)
Family parenting			0.038 (0.059)	0.099 (0.062)	0.015 (0.065)	0.080 (0.066)
Family education participation			0.082 *** (0.017)	0.058 *** (0.018)	0.078 *** (0.019)	0.055 *** (0.020)
Family lifestyle			0.006 (0.023)	−0.019 (0.025)	−0.004 (0.026)	−0.026 (0.027)
Family occupation			−0.015 (0.034)	0.010 (0.035)	−0.014 (0.038)	0.011 (0.038)
Family information			−0.001 (0.033)	0.021 (0.035)	−0.001 (0.037)	0.021 (0.038)
Family human expenditure			−0.013 (0.009)	−0.014 (0.009)	−0.007 (0.010)	−0.009 (0.011)
Family social communication			0.045 *** (0.012)	0.038 *** (0.012)	0.048 *** (0.013)	0.039 *** (0.013)
Medical insurance/Mediv			−0.004 (0.065)	−0.064 (0.068)	−1.427 *** (0.466)	−1.273 *** (0.476)
Endowment insurance			0.033 (0.034)	0.016 (0.036)	0.229 *** (0.076)	0.183** (0.078)
Government support			0.014 (0.039)	0.008 (0.041)	0.043 (0.045)	0.033 (0.045)
Tap water			0.089 ** (0.043)	0.058 (0.045)	0.091 * (0.048)	0.060 (0.049)
Fuel			0.048 (0.045)	−0.040 (0.048)	−0.003 (0.052)	−0.079 (0.053)
Air purification			−0.069 (0.102)	0.037 (0.108)	−0.073 (0.113)	0.034 (0.115)
R^2^	0.062	0.081	0.121	0.139	−0.064	0.021
SER	0.949	1.000	0.921	0.971	1.014	1.035
F					30.984	30.984

Note: *, **, and *** indicate significance at 10%, 5%, and 1% level, respectively; the standard error is in brackets under the coefficient.

## Data Availability

Data used in this paper can be found from the China Family Panel Studies, http://www.isss.pku.edu.cn/cfps/ (accessed on 13 March 2022).

## Appendix A

**Table A1:** Results for robustness tests.

	Robust 1 *N* = 2647	Robust 2 *N* = 2647	Robust 3 *N* = 2133	Robust 4 *N* = 2133	Robust 5 *N* = 2133	Robust 6 *N* = 2133
Intercept term	2.869 *** (0.648)	3.026 *** (0.661)	2.810 *** (0.680)	3.020 *** (0.693)	2.748 *** (0.688)	2.998 *** (0.701)
Child’s age	−0.055 *** (0.009)	−0.098 *** (0.009)	−0.050 *** (0.009)	−0.101 *** (0.010)	−0.050 *** ((0.009)	−0.102 *** (0.010)
Child’s gender	−0.284 *** (0.040)	0.003 (0.041)	−0.298 *** (0.045)	−0.004 (0.046)	−0.299 *** (0.045)	0.004 (0.046)
Child’s nationality	−0.476 (0.461)	−0.772 (0.470)	−0.408 (0.457)	−0.702 (0.466)	−0.446 (0.459)	−0.750 (0.468)
Family age	0.007 ** (0.003)	0.009 *** (0.003)	0.007 ** (0.003)	0.010 *** (0.003)	0.008 ** (0.003)	0.011 *** (0.003)
Family gender	−0.036 (0.046)	−0.069 (0.047)	−0.016 (0.051)	−0.045 (0.051)	−0.022 (0.051)	−0.053 (0.052)
Residence	−0.130 * (0.074)	−0.119 (0.075)	−0.113 (0.081)	−0.080 (0.082)	−0.116 (0.081)	−0.084 (0.082)
Urban–rural	−0.056 (0.049)	0.016 (0.050)	−0.070 (0.053)	0.030 (0.054)	−0.065 (0.054)	−0.024 (0.055)
Family marriage	0.037 (0.101)	0.037 (0.103)	0.085 (0.108)	0.046 (0.110)	0.082 (0.108)	0.047 (0.110)
Family size	−0.008 (0.011)	−0.011 (0.011)	0.000 (0.012)	−0.002 (0.012)	0.001 (0.012)	−0.001 (0.013)
Family cognitive ability	−0.021 (0.016)	−0.016 (0.016)	−0.018 (0.017)	−0.017 (0.018)	0.017 (0.018)	−0.016 (0.018)
Family income	−0.003 (0.022)	0.021 (0.023)	−0.010 (0.025)	0.013 (0.026)	−0.012 (0.025)	0.011 (0.026)
Children’s health investment	−0.002 (0.007)	−0.001 (0.007)	−0.001 (0.008)	−0.006 (0.008)	0.000 (0.008)	−0.004 (0.008)
Children’s education investment	0.014 (0.012)	−0.001 (0.013)	0.014 (0.014)	−0.006 (0.014)	0.014 (0.014)	0.006 (0.014)
Family education	0.086 *** (0.017)	0.089 *** (0.017)	0.080 *** (0.018)	0.090 *** (0.018)	0.079 *** (0.018)	0.088 *** (0.019)
Family education expectation	0.115 *** (0.021)	0.157 *** (0.022)	0.126 *** (0.023)	0.174 *** (0.024)	0.125 *** (0.023)	0.173 *** (0.024)
Family books	0.105 ** (0.046)	0.093 ** (0.046)	0.094 * (0.051)	0.084 (0.052)	0.097 * (0.051)	0.089 * (0.052)
Family parenting	0.012 (0.065)	0.075 (0.067)	0.008 (0.072)	0.072 (0.073)	0.004 (0.072)	0.065 (0.074)
Family education participation	0.076 *** (0.019)	0.054 *** (0.020)	0.089 *** (0.021)	0.066 *** (0.022)	0.087 *** (0.021)	0.064 *** (0.022)
Family lifestyle	−0.005 (0.026)	−0.028 (0.027)	0.007 (0.029)	−0.032 (0.030)	0.006 (0.029)	−0.034 (0.030)
Family occupation	−0.013 (0.038)	0.010 (0.039)	−0.015 (0.042)	0.007 (0.043)	−0.014 (0.042)	0.005 (0.043)
Family information	−0.002 (0.038)	0.020 (0.038)	0.007 (0.041)	0.010 (0.042)	0.006 (0.041)	0.011 (0.042)
Family human expenditure	−0.006 (0.010)	−0.008 (0.011)	−0.012 (0.011)	−0.010 (0.011)	−0.012 (0.011)	−0.010 (0.011)
Family social communication	0.044 *** (0.013)	0.035 *** (0.013)	0.046 *** (0.014)	0.042 *** (0.015)	0.042 *** (0.014)	0.036** (0.015)
Medical insurance	−1.450 *** (0.467)	−1.287 *** (0.477)	−1.447 *** (0.517)	−1.263 ** (0.527)	−1.474 *** (0.520)	−1.287 ** (0.530)
Endowment insurance	0.236 *** (0.076)	0.188 ** (0.078)	0.233 *** (0.082)	0.187 ** (0.084)	0.241 *** (0.083)	0.194 ** (0.085)
Government support	0.050 (0.045)	0.039 (0.046)	0.074 (0.049)	0.041 (0.050)	0.079 (0.049)	0.047 (0.050)
Tap water	0.092 * (0.048)	0.061 (0.049)	0.092 * (0.053)	0.064 (0.054)	0.092 * (0.053)	0.064 (0.055)
Fuel	−0.000 (0.052)	−0.082 (0.053)	0.040 (0.057)	−0.066 (0.058)	0.038 (0.057)	−0.069 (0.058)
Air purification	−0.076 (0.113)	0.028 (0.116)	−0.157 (0.128)	−0.098 (0.130)	−0.156 (0.128)	−0.099 (0.131)
Family relationship	0.008 (0.011)	0.001 (0.011)			−0.004 (0.012)	−0.004 (0.012)
Family health	0.038 ** (0.018)	0.041 ** (0.018)			0.036 * (0.020)	0.043 ** (0.020)
R^2^	−0.069	0.019	−0.050	0.041	−0.056	0.037
SER	1.017	1.037	1.008	1.027	1.011	1.029

Note: *, **, and *** indicate significance at 10%, 5%, and 1% level, respectively; the standard error is in brackets under the coefficient.

**Table A2:** Results for two-stage least squares by gender.

	Chinese		Math	
	Boy *N* = 1429	Girl *N* = 1218	Boy *N* = 1429	Girl *N* = 1218
Intercept term	2.049 * (1.158)	2.939 *** (0.941)	1.763 (1.185)	3.517 *** (0.949)
Child’s age	−0.060 *** (0.011)	−0.051 *** (0.014)	−0.097 *** (0.012)	−0.097 *** (0.014)
Child’s nationality	0.430 (1.013)	−0.683 (0.550)	0.571 (1.037)	−1.111 ** (0.554)
Family age	0.005 (0.004)	0.006 (0.004)	0.006 * (0.004)	0.009 ** (0.004)
Family gender	−0.049 (0.060)	0.002 (0.074)	−0.103 * (0.061)	−0.018 (0.075)
Residence	−0.071 (0.098)	−0.177 (0.114)	−0.066 (0.100)	−0.171 (0.115)
Urban–rural	−0.049 (0.067)	−0.067 (0.076)	−0.053 (0.068)	0.095 (0.076)
Family marriage	−0.108 (0.134)	0.214 (0.159)	−0.049 (0.137)	0.122 (0.160)
Family size	−0.019 (0.015)	0.007 (0.018)	−0.027 * (0.016)	0.007 (0.018)
Family cognitive ability	−0.009 (0.021)	−0.035 (0.025)	−0.003 (0.021)	−0.031 (0.025)
Family income	−0.002 (0.031)	0.004 (0.034)	0.019 (0.031)	0.035 (0.034)
Children’s health investment	−0.009 (0.009)	0.006 (0.012)	−0.006 (0.010)	0.001 (0.012)
Children’s education investment	0.016 (0.017)	0.009 (0.021)	0.011 (0.017)	−0.020 (0.021)
Family education	0.077 *** (0.022)	0.100 *** (0.027)	0.080 *** (0.022)	0.102 *** (0.027)
Family education expectation	0.093 *** (0.027)	0.157 *** (0.038)	0.162 *** (0.027)	0.157 *** (0.039)
Family books	0.076 (0.059)	0.135* (0.076)	0.088 (0.061)	0.095 (0.076)
Family parenting	0.110 (0.085)	−0.123 (0.108)	0.233 *** (0.087)	−0.117 (0.108)
Family education participation	0.035 (0.025)	0.133 *** (0.030)	0.015 (0.026)	0.104 *** (0.031)
Family lifestyle	−0.012 (0.035)	0.017 (0.041)	−0.038 (0.036)	−0.007 (0.041)
Family occupation	0.048 (0.052)	−0.053 (0.058)	0.062 (0.053)	−0.028 (0.058)
Family information	0.039 (0.051)	−0.066 (0.062)	0.056 (0.052)	−0.025 (0.063)
Family human expenditure	−0.010 (0.014)	−0.006 (0.016)	−0.015 (0.014)	−0.002 (0.016)
Family social communication	0.040 ** (0.017)	0.054 *** (0.021)	0.035 ** (0.017)	0.049 ** (0.021)
Medical insurance	−1.124 ** (0.560)	−1.958 ** (0.819)	−1.151 ** (0.573)	−1.619 ** (0.825)
Endowment insurance	0.186 ** (0.090)	0.298 ** (0.134)	0.127 (0.092)	0.271 ** (0.135)
Government support	0.021 (0.057)	0.089 (0.076)	0.060 (0.058)	0.004 (0.077)
Tap water	0.074 (0.064)	0.098 (0.077)	0.145 ** (0.065)	−0.042 (0.077)
Fuel	−0.003 (0.068)	−0.002 (0.082)	−0.074 (0.070)	−0.090 (0.083)
Air purification	0.082 (0.155)	−0.271 (0.174)	0.108 (0.158)	−0.080 (0.175)
R^2^	−0.011	−0.227	0.072	−0.058
SER	0.987	1.078	1.010	1.086

Note: *, **, and *** indicate significance at 10%, 5%, and 1% level, respectively; the standard error is in brackets under the coefficient.

**Table A3:** Results for two-stage least squares by urban location.

	Chinese		Math	
	Urban *N* = 1141	Rural *N* = 1506	Urban *N* = 1141	Rural *N* = 1506
Intercept term	2.069 ** (0.897)	3.458 *** (1.123)	1.815 * (0.940)	4.385 *** (1.179)
Child’s age	−0.059 *** (0.013)	−0.054 *** (0.012)	−0.087 *** (0.013)	−0.104 *** (0.012)
Child’s gender	−0.256 *** (0.059)	−0.318 *** (0.054)	−0.018 (0.062)	0.011 (0.057)
Child’s nationality	−0.404 (0.716)	−0.445 (0.606)	−0.300 (0.750)	−1.014 (0.637)
Family age	0.008 * (0.005)	0.004 (0.003)	0.012** (0.005)	0.006 (0.004)
Family gender	−0.086 (0.068)	0.009 (0.063)	−0.044 (0.071)	−0.065 (0.066)
Residence	−0.171 * (0.089)	−0.015 (0.163)	−0.179 * (0.093)	0.049 (0.171)
Family marriage	0.047 (0.151)	0.079 (0.137)	0.068 (0.158)	0.019 (0.144)
Family size	−0.010 (0.017)	−0.011 (0.014)	−0.004 (0.018)	−0.019 (0.015)
Family cognitive ability	−0.005 (0.022)	−0.029 (0.022)	0.004 (0.023)	−0.031 (0.023)
Family income	0.050 (0.032)	−0.040 (0.031)	0.059 * (0.034)	−0.002 (0.033)
Children’s health investment	−0.006 (0.011)	−0.001 (0.010)	0.010 (0.011)	−0.011 (0.011)
Children’s education investment	0.002 (0.019)	0.023 (0.016)	0.002 (0.020)	−0.002 (0.017)
Family education	0.065 ** (0.027)	0.101 *** (0.025)	0.069 ** (0.029)	0.116 *** (0.026)
Family education expectation	0.123 *** (0.034)	0.112 *** (0.028)	0.110 *** (0.035)	0.191 *** (0.030)
Family books	0.087 (0.062)	0.093 (0.069)	0.108 * (0.065)	0.054 (0.072)
Family parenting	0.100 (0.101)	−0.026 (0.086)	0.120 (0.106)	0.055 (0.090)
Family education participation	0.054 * (0.031)	0.092 *** (0.025)	0.055 * (0.032)	0.058 ** (0.026)
Family lifestyle	0.036 (0.038)	−0.028 (0.037)	−0.032 (0.039)	−0.010 (0.039)
Family occupation	0.061 (0.051)	−0.097 * (0.056)	0.064 (0.053)	−0.067 (0.059)
Family information	0.033 (0.055)	−0.020 (0.052)	0.042 (0.058)	0.006 (0.055)
Family human expenditure	−0.008 (0.015)	−0.005 (0.014)	−0.010 (0.016)	−0.009 (0.015)
Family social communication	0.041 ** (0.020)	0.057 *** (0.017)	0.035 * (0.021)	0.039 ** (0.018)
Medical insurance	−1.468 *** (0.478)	−1.300 (1.126)	−1.087 ** (0.501)	−1.861 (1.181)
Endowment insurance	0.243 ** (0.096)	0.200 (0.140)	0.193 * (0.100)	0.198 (0.147)
Government support	0.070 (0.067)	0.040 (0.067)	0.105 (0.070)	0.016 (0.071)
Tap water	0.149 * (0.084)	0.069 (0.064)	0.094 (0.089)	0.083 (0.067)
Fuel	0.097 (0.100)	−0.004 (0.067)	−0.060 (0.105)	−0.075 (0.071)
Air purification	−0.110 (0.131)	0.094 (0.220)	0.018 (0.138)	0.072 (0.231)
R^2^	−0.059	−0.035	0.018	−0.043
SER	0.978	1.030	1.025	1.081

Note: *, **, and *** indicate significance at 10%, 5%, and 1% level, respectively; the standard error is in brackets under the coefficient.
